# Wound Fluid Matrix Metalloproteinase-9 as a Potential Predictive Marker for the Poor Healing Outcome in Diabetic Foot Ulcers

**DOI:** 10.1155/2018/1631325

**Published:** 2018-10-16

**Authors:** Punyanuch Jindatanmanusan, Sivat Luanraksa, Tanit Boonsiri, Thirayost Nimmanon, Pasra Arnutti

**Affiliations:** ^1^Department of Pediatrics, Phramongkutklao College of Medicine, Bangkok, 10400, Thailand; ^2^Division of Plastic and Reconstructive Surgery, Lerdsin Hospital, Bangkok, 10500, Thailand; ^3^Department of Microbiology, Phramongkutklao College of Medicine, Bangkok, 10400, Thailand; ^4^Department of Pathology, Phramongkutklao College of Medicine, Bangkok, 10400, Thailand

## Abstract

**Background and Objective:**

Evidence for the roles of matrix metalloproteinases-9 (MMP-9) in the healing process of diabetic foot ulcers has remained unclear. We therefore aimed to demonstrate the relationship of MMP-9 with the wound healing process and determine its potential usefulness in predicting the wound healing outcome.

**Methods:**

Twenty-two patients with diabetic foot ulcer were recruited. The wound size was determined, and the wound fluid was collected for the measurement of MMP-9 levels using an ELISA during the 12-week follow-up period regularly. The patients were categorized as good healers and poor healers when the wound area reduction was ≥ 50% and < 50% at week 4 when compared to the initial wound size at week 0.

**Results:**

Median wound fluid MMP-9 levels in the poor healer group were shown to be significantly higher than those in the good healer group (1.03 pg/*µ*g protein vs. 0.06 pg/*µ*g protein, p = 0.001), and the levels fluctuated throughout the 12-week follow-up period. In contrast to the poor healer group, the MMP-9 levels were demonstrated to be constantly low throughout the follow-up period in the good healer group. ROC analysis showed that the MMP-9 level of 0.38 pg/*µ*g protein was able to predict the wound healing outcome with the sensitivity of 81.8%, the specificity of 64.6%, and the area under the curve of 0.901 (CI 0.78-1.03, p = 0.001).

**Conclusion:**

These findings suggested that determination of wound fluid MMP-9 levels might become a promising biomarker predicting wound healing outcomes and a novel potential therapeutic target for diabetic foot ulcers.

## 1. Introduction

Wound healing is a complex process that can lead to chronic ulcers if any stage in this intricate series of events is interrupted [1]. Like other types of chronic ulcers, which result from an exaggeration of the inflammatory phase, diabetic ulcers have a prolonged inflammatory phase along with significant increases in proinflammatory cytokines, proteases (MMPs), and neutrophil elastase [2]. The increases in these protein levels are believed to be responsible for the increased extracellular matrix destruction observed in chronic wounds [3]. Furthermore, the excess of MMPs in chronic wounds is proposed to be contributory to poor wound healing through their action in breaking down of many extracellular matrix components and in inhibiting growth factors that are essential for tissue synthesis and wound healing [4, 5]. Collectively, these effects of high MMP levels result in poor wound healing efficiency.

Several studies have shown that high MMP-9 levels have a significantly deleterious effect on diabetic foot ulcer healing even though the mechanism of the increase is still uncertain [6–10]. MMP-9 has been related to increased inflammation, given that MMP-9 is expressed mainly by neutrophils and macrophages, and both these cell types are crucially important for the inflammatory response [11]. This pattern of increased MMP-9 in poorly healing ulcers was observed in varying types of diabetic foot ulcers, suggesting that it is more strongly linked with the healing process rather than with an underlying etiology. However, the studies on human diabetic foot ulcers are limited by the difficulty in obtaining tissue samples [7–9]. As a result, relatively few studies have determined MMP activity in wound fluid specifically from diabetic foot ulcers, and data concerning the dynamic change in MMP-9 levels during the healing of chronic diabetic foot ulcers are rather limited. This study therefore aimed to determine the relationship between MMP-9 concentrations and the healing process of chronic diabetic wounds and to prove whether MMP-9 levels can become a potential predictive biomarker of the poor wound healing outcome.

## 2. Materials and Methods

Twenty-two patients with type 2 diabetes were recruited in the study at the Plastic and Reconstructive Surgery Department, the Lerdsin Hospital. Each patient voluntarily gave informed consent to participate in the study, which was approved by the Ethical Committee of Lerdsin Hospital (IRB 571024). All patients had a chronic wound with a duration ranging from 1 month to 24 months and with an area of at least 0.25 cm^2^. The ulcers were graded on a 1-to-3 scale according to the Texas Grading System [5]. All patients received local wound care and offloading treatments every day during the 12-week study period and until all wounds were healed, following the standard protocol for diabetic foot ulcers that had been used in our department. Exclusion criteria include conditions potentially interfering with MMP levels, consisting of arteriopathy of the lower limb, infection, and use of any substance that may disturb MMP levels, such as Promogran [12]. The arteriopathy of the lower limb was characterized by either by the absence of posterior tibial and pedal pulses or by an ankle/brachial index of <0.9.

The wound area was measured at the each visit (first visit or week 0, week 1, week 4, week 8, and week 12) using the gridded paper (OPSITE◊FLEXIGRID◊, smith&nephew, UK) as a size reference. All patients were divided into two subgroups according to the rate of wound healing at week 4 when compared to week 0. Patients with a decrease in wound area of at least 50% were defined as the ‘good healers', whereas those with a decrease in wound area of less than 50% were defined as the ‘poor healers' [13]. Wound fluid samples were collected at the each visit by applying a sterile absorbent paper strip (Schirmer strips, Alcon®, Canada) over the wound for 5 minutes. Proteins were eluted from the paper strip by refrigerated centrifugation in 1 ml buffer (50 mM Tris, 50 mM NaCl, 0.05% Brij 35, pH 7.6) for at least 2 hours at +4°C. After the strip was removed, the protein solution was stored at -20°C until any protein and enzyme analyses were performed at the laboratory of the Department of Pathology, Phramongkutklao College of Medicine [9, 14]. MMP-9 levels were measured using an enzyme-linked immunosorbent assay (ELISA, Abcam®, UK). To avoid any possible variations caused by the amount of fluid collected, protein concentrations were measured using an enzymatic method (COBAS c501 analyzer, Roche® Diagnostics, USA) and expressed as pg/*µ*g of total protein. Wound fluid collection was discontinued when the wound was completely healed.

Statistical analysis was performed using statistical software, STATA/MP 13 for Windows7. Mann–Whitney or Wilcoxon test, Chi-squared, or Fisher tests were used for nonparametric tests as appropriate. The Spearman test was performed to detect correlations between percentages of wound area reduction and changes in MMP-9 levels. A threshold of *α*≥0.05% was used for all statistical tests. Receiver Operator Curve (ROC) analysis was used to determine the cut-off level of MMP-9 concentrations with respect to wound healing.

## 3. Results

**Figure 1:**
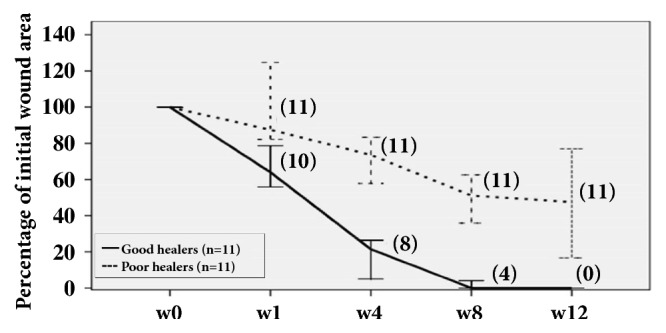
Percentages of initial wound surface areas in good healer and poor healer groups are displayed as median with interquartile range (25th-75th percentile). By week 8, wounds in 7 out of 11 good healers were healed, whereas no wound in the poor healer group was healed during the 12-week study period.

All of patients were followed up for 12 weeks unless their wound was healed completely before the end of the study. Among 22 patients, 11 patients were defined as good healers, whereas the rest were defined as poor healers. All the wounds in the good healer group were completely healed before week 12, whereas none of the wounds in the poor healer group were healed throughout the 22-week study period. The dynamic change in wound surface area in both groups is shown in Figure 1, and the demographic and clinicopathologic data are summarized in Table 1. There was no statistical difference in age, duration of wound, HbA1c, ABi, or initial size of wound between the good healer and poor healer groups.

### 3.1. Dynamic Change of MMP-9 during the Wound-Healing Process

**Figure 2:**
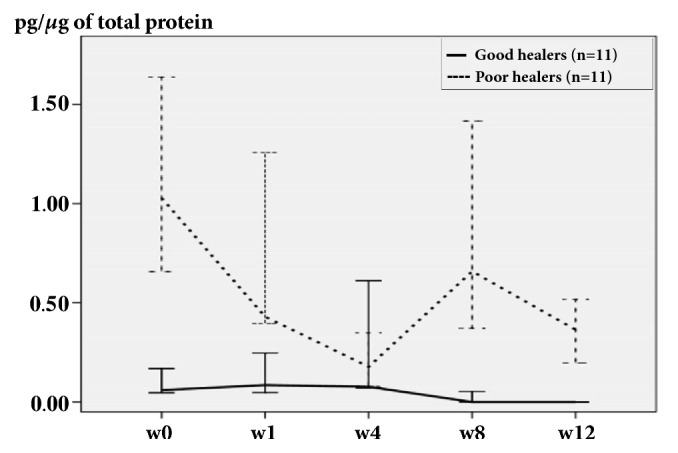
MMP-9 levels in good healer and poor healer groups during the 12-week follow-up period are expressed as median with interquartile range (25th-75th percentile).

In poor healers, MMP-9 levels were higher than those in the good healer group at W0 (p=0.001). The levels in good healers were constantly low. In contrast, the levels in poor healers fluctuated throughout the 12-week study period, revealing a decrease at week 4, an increase at week 8, and another decrease at week 12, but these changes revealed no statistical significance (Figure 2). Noteworthy, because wounds were already healed in many good healers at week 8 and 12, only 4 samples were collected at week 8 and no collection was performed at week 12 in the good healer group.

### 3.2. Predictors of Wound Healing

**Figure 3:**
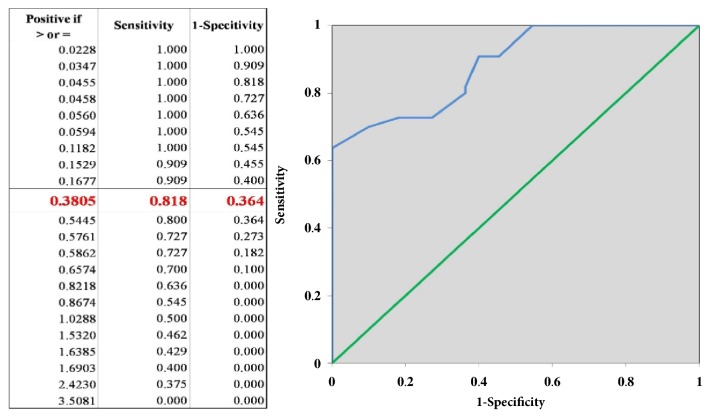
The MMP-9 is a predictive factor for diabetic ulcer wound healing outcome. The ROC analysis gives an area under the curve of 0.90(0.6–1.04). The MMP-9 level of 0.3805 at week 0 has a sensitivity of 81.8% and a specificity of 64.6% for detecting a wound area reduction of less than 50% at week 4.

Initial MMP-9 levels at week 0 were negatively correlated with percentages of reduction in wound area at week 4 (r = -0.5249, p = 0.006), thereby being well correlated with the wound classification into good and poor healer groups. According to the ROC analysis, initial MMP-9 levels at week 0 were shown to be a prognostic indicator of good/poor healing during the 12-week follow-up period (Figure 3).

## 4. Discussion

Agreeing with many previous studies, we found that MMP-9 concentrations were higher in exudates of unhealed wounds than healed wound, even though we selectively focused only on the diabetic population [6–10]. Many factors capable of interfering with MMP levels were eliminated by carefully selecting diabetic patients who did not have arteriopathy, infection, or use of any substances that may disturb MMP levels. The dynamic expression of MMP-9 levels during the healing process was also described in both good and poor healer groups. Importantly, using ROC analysis, we proposed the cut-off level for MMP-9 at 0.38 pg/*µ*g of total protein. Using this cut-off level, the healing outcome could be predicted with the sensitivity of 81.8%, the specificity of 64.6%, and the area under the ROC curve of 0.901.

In normal circumstances, a small amount of MMP-9 is secreted in the inflammatory phase in which the healing process proceeds, allowing the progression of the healing process to the proliferation phase. The high MMP-9 levels in the poor healer group support the idea that an excess of some MMPs, particularly those secreted by inflammatory cells, could have a deleterious effect on the healing process. Not surprisingly, this enzyme was shown to be increased in the poor healer group when compared to the good healer group. Furthermore, the presence of high MMP-9 levels also suggested the ongoing inflammatory phase, given that this enzyme is expressed by the 2 important inflammatory cells, neutrophils and macrophages, which play important roles in the inflammatory process [11]. In spite of the established association between high MMP-9 levels and poor wound healing, the mechanism of increased MMP-9 levels is still uncertain [6], even though the direct deleterious effect of the enzyme on re-epithelialization, a process required for wound healing, has recently been proposed in a murine wound model [15]. Regardless of the rather unknown actual mechanism of MMP-9 in prevention of wound healing, interfering with this enzyme as well as other MMPs by a selective dressing method has been shown to benefit patients with difficult-to-treat ulcers [16, 17]. This strategy might especially be crucial for those with MMP profiles compatible with the poor healer group, including a high MMP-9 level.

Currently, there is no consensus method for the measurement of MMP activity in wound fluid. Samples were collected using absorbent strips, which absorb the fluid by capillary action. This sample collection procedure can be performed in outpatient settings because of its rapidity, simplicity, and noninvasiveness. Furthermore, this collection method was selected, because by using it, local expression of MMPs in wound fluid, which better reflects the microenvironment of the wound than their expression in the serum, could be directly determined. MMP concentrations in our study were shown to be comparable to those in other previous studies that examined the levels in would fluid and expressed the values in the same unit (pg/*μ*g of protein) [18]. Noteworthy, we analyzed MMP-9 levels using an ELISA technique, in contrast to many previous studies on MMP-9, which determined its levels using a zymography technique [11]. Even though the zymography technique is a useful qualitative technique, it lacks specificity and true quantitative nature, which could be obtained utilizing an antibody-based activity assay, such as ELISA [19]. Nonetheless, the ELISA technique detects both active and inactive forms of the enzyme and may not reflect actual enzyme activity in the wounds, since the enzyme is extracellular activated after its production in an inactive form [20]. To precisely relate the MMP-9 activity to the wound healing outcome, commercially available kits or any other quantitative methods that specifically detect the activated form of MMP-9 are required.

The results in this study might have been interfered by several factors. Firstly, the number of patients in this study was relatively small, thus the relatively low statistical power of our results. Secondly, despite the stringent inclusion criteria, diabetic foot ulcers were heterogeneous in nature at least partly due to the high prevalence on medical calcinosis in diabetic vessels. On the contrary, the stringent criteria themselves might also partially limit data generalization given that this did not ideally reflect the real life situation with diverse clinical settings. Thirdly, different ulcer locations might have affected the prognosis of wound healing, making the prediction of wound healing outcome difficult. Finally, since the investigating laboratory was located in a different institute from the sample collection site, the temperature- and time-dependent denaturation could also influence the protein level assessment, even though cooling box was used for the temperature control during the delivery.

## 5. Conclusion

The wound fluid levels of MMP-9 at presentation (week 0) might be able to predict the healing outcome and suggest the therapeutic strategy tailored to individual patients with diabetic foot ulcers. Furthermore, levels of other enzymes, such as other MMPs, TIMP-1, and TGF-*β*1, might further enhance the prognostic value of MMP-9 levels when measured together at presentation.

## Figures and Tables

**Table 1:** Characteristics of patients at inclusion.

	Good healers (N=11)	Poor healers (N=11)
Gender (M/F)	7/4	8/3
Age (years)	58 (35-79)	55 (46-65)
Wound surface area (cm^2^)	3.5 (0.25-12.5)	6.75 (1.05-31.25)
Wound Duration (months)	4 (1-12)	4 (1-24)
HbA1C (mg%)	7.6 (6.4-9.6)	7.4 (6.8-11.8)
ABi	1.2 (1.1-1.7)	1.18 (0.84-1.48)
MMP-9	0.06 (0.02-0.82)	1.03 (0.12-3.50)

HbA1C: glycated hemoglobin; ABi: ankle brachial index; MMP: matrix metalloproteinase. Values are expressed as median with interquartile range (25^th^-75^th^ percentile).

## Data Availability

The data of MMP9 levels used to support the findings of this study are available from the corresponding author upon request.
